# Giant thrombus in aortic arch and pulmonary embolism

**DOI:** 10.1016/j.jvscit.2026.102359

**Published:** 2026-06-23

**Authors:** Takayuki Gyoten, Akihiro Yoshitake

**Affiliations:** Department of Cardiovascular Surgery, Saitama Medical University International Medical Center, Saitama, Japan

A 44-year-old man developed sudden right-sided hemiparesis 4 days after hospital admission. He was receiving heparin therapy for acute pulmonary embolism secondary to deep vein thrombosis (DVT) after a long flight. He had no significant medical history. Brain magnetic resonance imaging revealed an acute ischemic stroke in the left hemisphere. Computed tomography showed a large thrombus extending from the ostia of the left common carotid and left subclavian arteries to the descending aorta (*A* and *B*), consistent with embolic cerebral ischemia. Transthoracic echocardiography demonstrated a patent foramen ovale (PFO) with a 1.5-cm bidirectional shunt (Supplementary Video 1, online only). After discussion by the heart team, paradoxical embolism associated with DVT and pulmonary embolism was diagnosed. Urgent surgical thrombectomy was performed to reduce the risk of further embolic events from the large floating thrombus, which was refractory to heparin therapy. A fresh thrombus measuring 30 cm in length was directly removed through a 5-cm incision on the anterior aspect of the aortic arch under circulatory arrest (*C*/Cover). Pulmonary thrombectomy and direct PFO closure were then performed. Postoperative testing suggested an underlying protein C deficiency. At the 6-month follow-up, CT showed no residual thrombus. The patient remained free of neurologic complications and continued anticoagulation monotherapy. We report a rare case in which a thrombus originating from DVT was considered to have traversed a PFO into the aortic arch, with concomitant pulmonary embolism and secondary thrombus enlargement associated with underlying protein C deficiency.^1^^,^^2^ Written publication consent was obtained from the patient.

**Figure:**
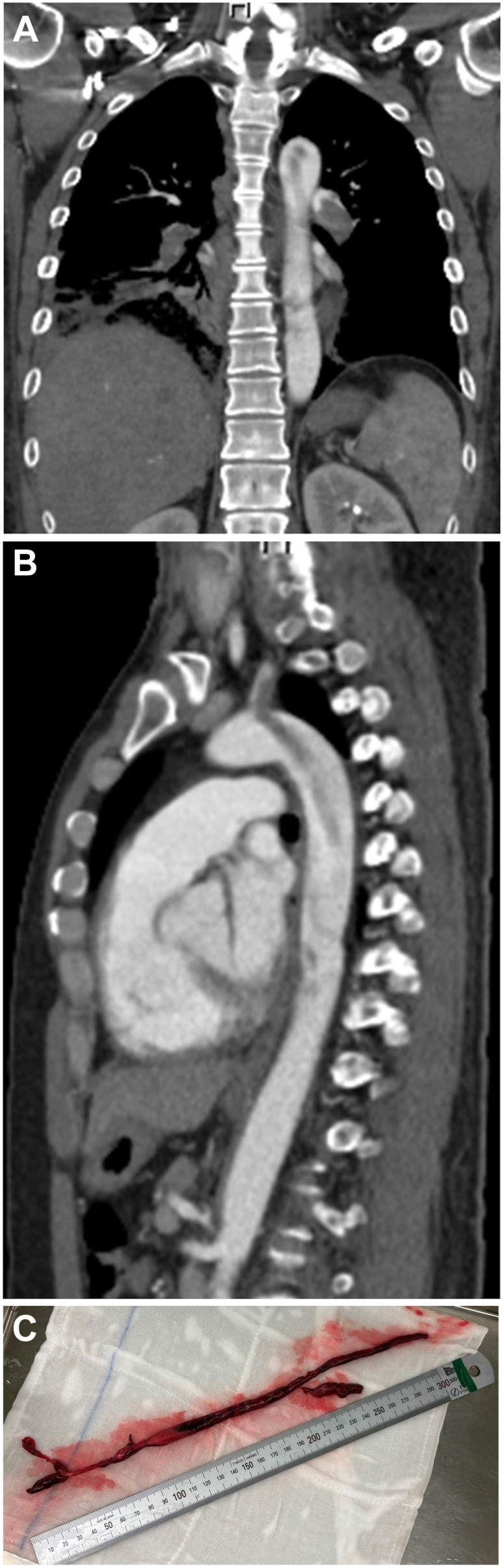

## Funding

None.

## Disclosures

None.

## Figures and Tables

**Cover Image:**
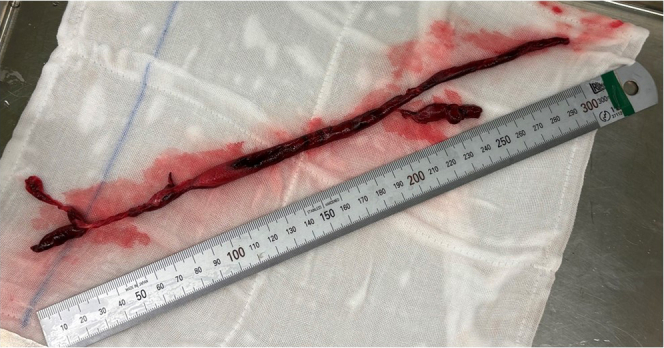


## Appendix

Supplementary Video 1 (online only) Transthoracic echocardiography demonstrated a patent foramen ovale.
